# Spatial transcriptomics of developing human lungs defines cellular phenotypes associated with age, lineage and location

**DOI:** 10.1038/s41598-025-34594-z

**Published:** 2026-01-03

**Authors:** Yue Ren, Soula Danopoulos, Gail H. Deutsch, Ian A. Glass, Thomas J. Mariani, Soumyaroop Bhattacharya

**Affiliations:** 1 Center for Children’s Health Research, Department of Pediatrics, School of Medicine and Dentistry, University of Rochester, NY Rochester, USA; 2 Lundquist Institute for Biomedical Innovation at Harbor-UCLA Medical Center, Torrance, CA USA; 3 Department of Laboratory Medicine and Pathology, University of Washington School of Medicine and Seattle Children’s Research Institute, Seattle, WA USA; 4 Department of Pediatrics, University of Washington School of Medicine, Seattle, WA USA; 5 Department of Pediatrics, David Geffen School of Medicine at UCLA, Los Angeles, CA USA

**Keywords:** Visium, Prenatal lung, Pseudoglandular, Canalicular, Deconvolution, Methylation, Cell cycle, Developmental biology, Molecular medicine, Transcriptomics

## Abstract

Despite significant advances in understanding lung development, the intricate cellular interactions and spatial organization of the developing human lung remain incompletely defined. Spatial transcriptomics enables gene expression profiling within the native tissue context, providing unprecedented insights into complex developmental processes. In this study, we applied the 10X Genomics Visium platform to characterize spatially resolved transcriptional profiles of prenatal human lungs during the pseudoglandular and canalicular stages.Spatial transcriptomic analysis of 12 prenatal lung samples (13–20 weeks gestation) identified 10 distinct transcriptional niches corresponding to unique combinations of epithelial, mesenchymal, endothelial, and immune cell populations. Unsupervised clustering revealed developmental shifts in spot/niche composition from the pseudoglandular to canalicular stage, with a progressive increase in alveolar epithelial spots and a concomitant decline in mesenchymal regions, particularly in peripheral lung areas. Differential gene expression analysis demonstrated stage-specific transcriptional transitions in individual spot types, including downregulation of cell cycle and structural pathways and upregulation of secretory pathways as the lung matures. Spatial organization analysis revealed increasing compartmentalization of pulmonary cell types, highlighting the progressive structuring of the distal lung microenvironment. In summary, this study provides a spatial map of the developing human lung, offering novel insights into pulmonary lineage dynamics and cellular interactions during early organogenesis.

**Supplementary Information:**

The online version contains supplementary material available at 10.1038/s41598-025-34594-z.

## **Introduction**

The mature respiratory system is exceptionally complex at the anatomical and cellular levels, and is comprised of multitude of different cell types^1^. The lung is a highly spatially complex organ, with intricate structural and cellular organization exemplified by the fractal-like branching of airways, from the trachea to millions of alveoli^2^. Human lung development progresses through several stages, starting in the early embryonic period and continuing throughout gestation and postnatally. In humans, the development of the lower respiratory tract, including the lungs initiates early (3 weeks of gestation) in the gestational process and continues until the child is eight years of age. This process is divided into five stages: embryonic (3–5 weeks of gestation), pseudoglandular (6–16 weeks of gestation), canalicular (17–26 weeks of gestation), saccular (27–36 weeks of gestation), and alveolar (36 weeks of gestation till eight years after birth) stages^3,4^. The development of the human lung involves complex, independent processes including branching, cell differentiation, and surfactant production. These processes can be conceptualized as occurring over time and in distinct spatial locations. Development and maintenance of lung structure requires cross talk among multiple cell types to coordinate lineage specification, cell proliferation, differentiation, migration, morphogenesis, and injury repair. The heterogeneity of cell types during embryonic lung development makes it difficult to study pulmonary fate decisions using traditional developmental biology techniques.

Recent advances in imaging and sequencing technologies have illuminated the fine-scale spatial architecture of the lung, deepening our understanding of its function in both health and disease^5^. We have previously leveraged bulk RNA sequencing to uncover key molecular processes driving normal lung development^6,7^ and associated pathologies^8–10^. More recently, single-cell RNA sequencing (scRNA-seq) has revolutionized lung biology by enabling high-resolution profiling of cellular diversity and gene expression dynamics.

The Human Lung Cell Atlas applied scRNA-seq to over 75,000 cells from human lung and blood tissues, revealing 58 molecularly distinct cell populations, including 14 previously unrecognized cell types^11^. Building on this, the uniLUNG project compiled an atlas of over 10 million cells from healthy and diseased lungs, identifying novel immune subsets such as Lym-monocytes and T-like B cells^12^. Application of scRNA-seq to fetal and neonatal lung tissues has enabled detailed characterization of cell types across developmental stages, offering insights into the molecular mechanisms of lung maturation in both normal and abnormal contexts^13–15^. In disease contexts, scRNA-seq has revealed critical alterations in cellular composition and function. Collectively, these studies underscore the transformative impact of single-cell technologies in unraveling the cellular and molecular complexity of the human lung in development and disease.

scRNA-Seq alone does not preserve spatial information, and does not enable studies of the complex interplay between cellular differentiation and morphogenesis^16^. Spatial transcriptomics is a relatively new technique that allows for the simultaneous analysis of gene expression and spatial location within tissues. It provides valuable insights into the molecular organization of tissues and has been applied to various research areas, including developmental biology and disease studies. Spatial transcriptomics has significantly advanced our understanding of the lung architecture and function by enabling high-resolution mapping of gene expression within tissue contexts. Stereo-seq analysis of a female mouse lung revealed distinct spatial expression patterns corresponding to various lung functions and identifying a gradient of proliferation-related gene expression along the proximal-to-distal tracheal axis^17^. A spatial map of the developing mouse lung identified ten critical spatial domains essential for lung organization and constructed lineage maps connecting spatial clusters across developmental stages, providing insights into transcription factor regulatory networks and signaling pathways involved in alveolar maturation^18^. A combinatorial approach of applying single cell sequencing with spatial transcriptomics identified 80 distinct cell types, including 11 previously unrecognized ones^19^. Spatial transcriptomics has been used to simultaneously capture host and pathogen gene expression in formalin-fixed paraffin-embedded lung tissues from COVID-19 subjects, enhancing our understanding of localized host-pathogen interactions^20^. While most of the studies applying spatial transcriptomics in lung tissues have either used animal models or adult human lungs^16^, there are limited publications describing spatial transcriptomics characterization of prenatal human lung development^21,22^. Unlike previous studies which profiled a broader gestational range^21,22^, our study analyzes a window of 13–20 weeks post-conception and includes the critical transition from pseudoglandular to canalicular stages of lung development, thereby enabling direct spatial comparisons across the developmental trajectory.

Here, we leverage the application of spatial transcriptomics to compare the ontogeny of cellular niches in human fetal lungs encompassing early stages of development, which will provide new insights into the spatial organization and maturation of the pulmonary microenvironment not captured by previous studies, and reconstruct a high-resolution, spatially resolved gene expression census of developmental pulmonary cellular lineages.

## **Results**

To localize developmental shifts in resident pulmonary cell types and to gain insight into their cellular neighborhood, we performed spatial transcriptomics using the 10X Genomics Visium platform. We profiled hematoxylin and eosin (H&E) stained tissue sections from twelve prenatal human lung samples ranging in gestational age from 13 to 20 weeks. Sequence data demultiplexing and pre-processing was completed using CellRanger and aligned to GRCh38. The samples yielded over the manufacturer recommended threshold of 50,000 mean reads per Spot, indicating good sample and data quality. Spots from all slides were integrated into one data object, representing 43,435 spots. To mitigate technical variability and batch effects inherent in spatial transcriptomic data from multiple samples, we integrated datasets using proven batch correction algorithms, enabling robust detection of shared developmental and spatial expression patterns across gestational stages. Samples with marginal tissue section quality (DV200 < 50%) had lower number of spots that passed the filtering thresholds. Upon removal of low-resolution spots based on the combination of multiple parameters including low gene and UMI counts, 28,335 (65%) spots, with 36,945 consistently expressed genes, were retained in the analytical dataset (Figure S1). The absence of distinct sample-specific clustering suggests that key transcriptional programs driving early lung development are maintained throughout mid-gestation (Figure S2 and Table S1).

### *Spot annotation*,* location*,* and composition in developing human lungs*

**Fig. 1:**
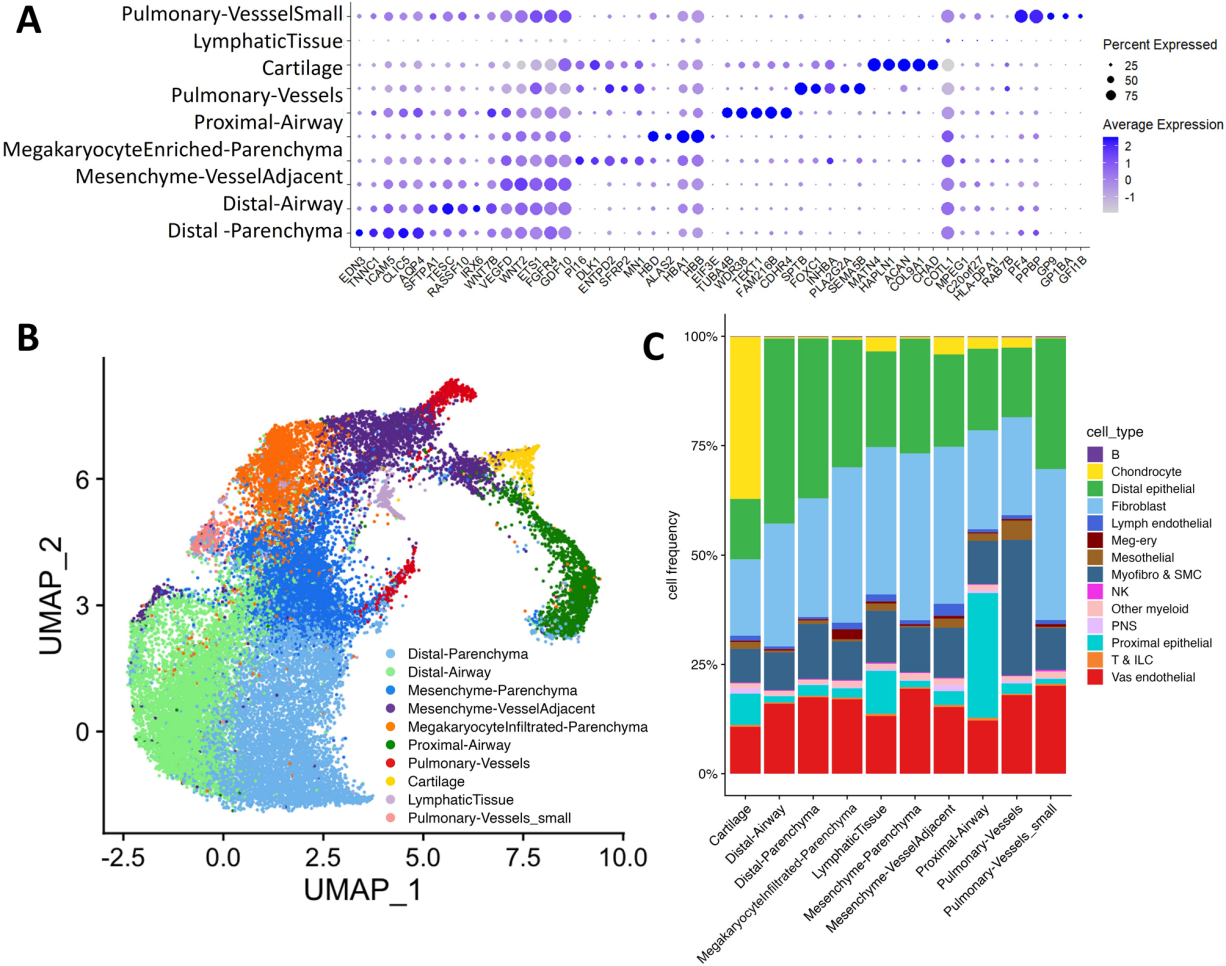
Identification and Annotation of Distinct Pulmonary Microenvironments. (**A**) Expression of Cluster Markers. Dot plot of markers used to identify annotations of the spots grouped into clusters. Within these populations the percentage of cells expressing the gene (using dot size) and the average expression level based on unique molecular identifier (UMI) counts (depth of color) of. spots obtained from 12 prenatal human lung tissues. (**B**) Uniform Manifold Approximation and Projection (UMAP) displays the 10 unique spot clusters. Spots are colored by the tissue type annotation presented in the UMAP display in the legend. (**C**) Deconvolution Analysis. Shown here are the frequencies of the cellular identities of the reference dataset in each of the spot clusters in the human prenatal lung tissue samples.

Unsupervised clustering of the 28,335 high quality spots identified 10 distinct groups of prenatal pulmonary spots representing distinct combinations of epithelial, mesenchymal, endothelial and immune cells as indicated by the expression of major lineage markers (Figure S3). The 28,335 high quality spots were annotated based on the expression of major lineage markers (Figure S3). Marker genes for individual spot clusters were identified based upon selective expression (Fig. 1A). Functional enrichment analysis using multiple resources^19,21,23,24^ available through the ToppFun^25,26^, and PanglaoDB^27^, successfully identified the spot types for each of the 10 clusters (Fig. 1B). The spatial spot clustering reveals a clear separation of the major spatial domains within the developing lung. The distal parenchyma spots form a large and relatively cohesive cluster, indicating a shared transcriptional identity across these regions, whereas the distal airway and proximal airway spots clustered distinctly, reflecting the transcriptional differences between these airway compartments. Mesenchymal populations, including mesenchyme parenchyma and mesenchyme vessel adjacent, cluster distinctly, suggesting transcriptional heterogeneity within the developing mesenchyme. Spots associated with vascular structures, pulmonary vessels and pulmonary vessels small, also form separate clusters. The detection of megakaryocyte infiltrated parenchyma and lymphatic tissue spots indicate the presence of immune cells and developing lymphatic structures within the prenatal lung tissue. There is a small distinct cluster of cartilage which is consistent with their localized presence in the developing airways. The organization of spot clusters also provides insights into the transcriptional relationships between different tissue compartments, for instance, the proximity of mesenchyme parenchyma spots to the distal parenchyma spots may indicate potential interactions and signaling between these cell types during alveolar development.

We assessed the compositions of cells in each spot type using the RCTD algorithm^28^. Each spot was deconvoluted on the basis of the annotated scRNA-seq data of cell atlas of human lung development^21^. Comparison of the spot annotations determined by ToppCell Atlas and estimated cell type abundance confirmed successful mapping of well-described cell types to their expected location, such as arteries and veins or smooth muscle cells around large vessels, as well as ciliated and mucous cells in airways. Integrated expression profiles from scRNA-seq with spatial transcriptomics data revealed substantial consistency (30% or higher match) in annotated spot types and reference cell types, between spatial spot cluster annotations with the cellular identities derived from the reference dataset (Fig. 1C), validating the spatial transcriptomics approach for the analysis of prenatal lung tissue. Distal airway spots were determined to be composed of 42% distal epithelial cells, followed by 28% of fibroblasts, and 16% of vascular endothelial cells. Mesenchymal parenchyma spots were composed of 38% fibroblasts, followed by 22% distal epithelial cells, 19% vascular endothelial cells, and 10% myofibroblasts and smooth muscle cells. Distal parenchyma spots were composed of 37% distal epithelial cells, 27% fibroblasts, 17% vascular endothelial cells, and 12% myofibroblasts and smooth muscle cells. Proximal airway cells were composed of 29% proximal epithelial cells, 23% fibroblasts, 19% distal epithelial cells, and 12% vascular endothelial cells. Cartilage spots were composed of 37% chondrocytes, 17% fibroblasts, 14% distal epithelial cells, and 11% vascular endothelial cells. Pulmonary vessel spots were composed of 31% myofibroblasts & smooth muscle cells, 22% fibroblasts, 18% vascular endothelial cells, and 16% distal epithelial cells. Composition of each spot type is presented in Figure S5.

**Fig. 2:**
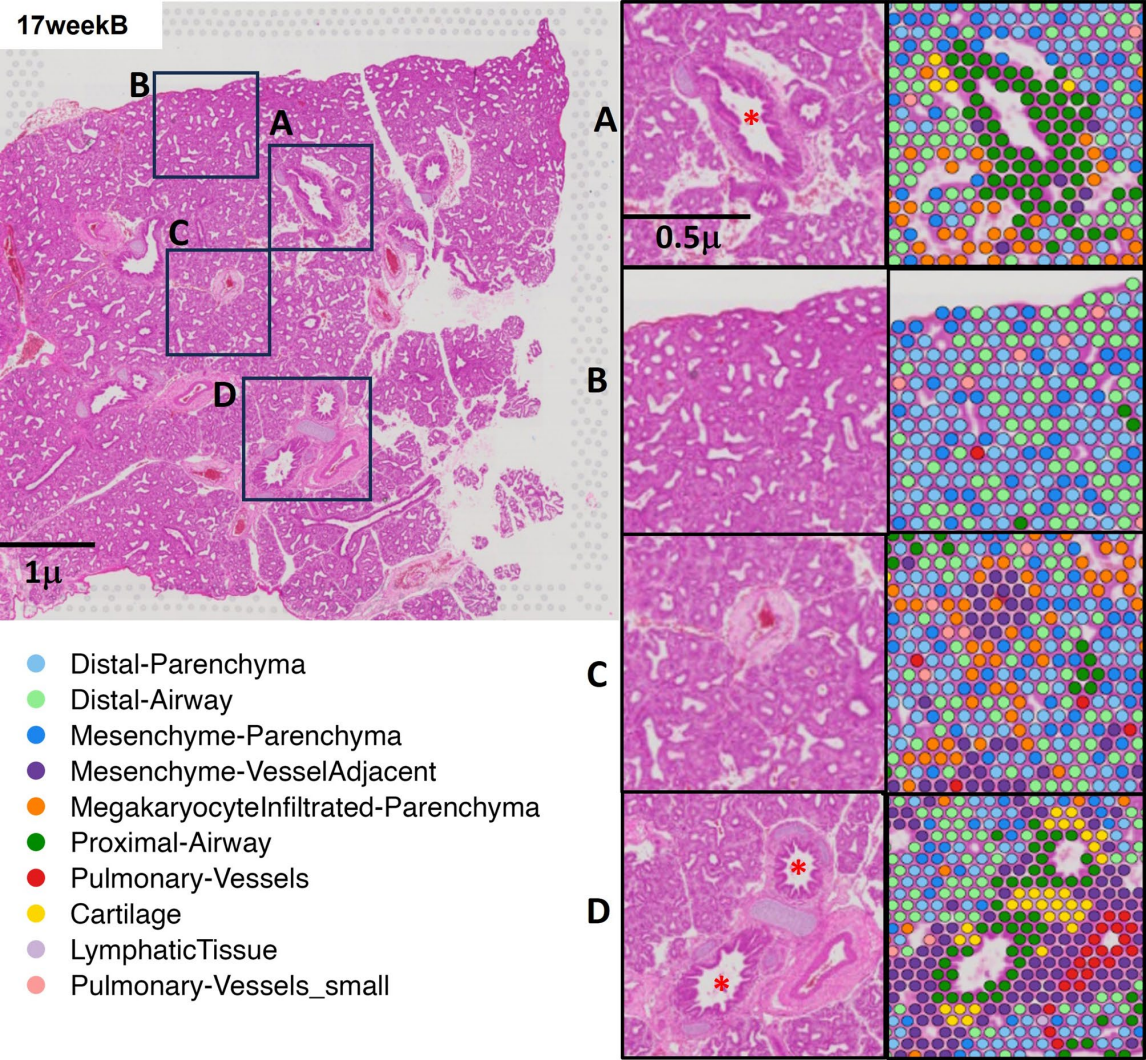
Spot types and their spatial locations: (**A**) human prenatal lung tissue section at 17 weeks gestation is shown with eosin staining. The left panel displays the full tissue section with four regions of interest (**A**–**D**) highlighted. The right panel presents high-magnification views of these regions, with corresponding spatial transcriptomic spot annotations overlayed. Each spot represents a defined transcriptomic region, color-coded according to the legend to indicate different tissue types. * Indicates airways.

This spatial transcriptomic approach enabled the molecular characterization of distinct lung microenvironments during development. Analysis of the locations of the spot clusters revealed spatial heterogeneity in the prenatal human lung tissue sample at 17 weeks of gestational age (Fig. 2). Specifically, inset A highlights a developing airway structure surrounded by distal parenchyma. The spatial spots in this region demonstrate a mixture of distal parenchyma and distal airway spots, reflecting the cellular heterogeneity at the interface of these compartments. Inset B focuses on a region enriched in mesenchyme adjacent to developing parenchymal tissue. The spatial spots in the corresponding segmented image are predominantly annotated as mesenchyme parenchyma, indicating the presence of mesenchymal cells near the developing epithelium. Inset C illustrates another developing airway. The spatial spots annotated as distal airways are centrally located within the airway structure, while surrounding spots show a transition to ‘Distal-Parenchyma. Finally, inset D showcases a region containing developing vascular structures embedded within the mesenchyme. The spatial spots in this inset reveal annotations corresponding to mesenchyme vessel adjacent near the spots annotated as pulmonary vessels and pulmonary vessels small, highlighting the spatial relationship between these cell types during vascular development.

Tissue composition across all samples indicated predominance of distal parenchyma, which corresponds to the developing alveolar regions of the lung, while mesenchymal parenchyma and mesenchyme adjacent to vessels are also widespread, highlighting the importance of mesenchymal interactions in lung development. An examination of vascular and airway structures showed presence of distal and proximal airways in varying degrees, suggesting differences in airway branching and maturation across samples, while pulmonary vessels, like arteries, and veins are distributed throughout, most likely corresponding to developing vasculature (Figure S6).

### *Developmental transition in spatial complexity*

Based on the range of gestational ages, the samples used in this study belong to early late pseudoglandular (13–16 weeks of gestation) to early canalicular (17–20 weeks of gestation) stages (Table 1). Early timepoints were characterized by large, relatively homogenous domains of mesenchymal and distal epithelial tissue, whereas later timepoints showed increased spatial complexity with the emergence of discrete vascular, immune, and airway compartments. In the lung tissues corresponding to the pseudoglandular stage (*n* = 6), we observed greater heterogeneity in tissue organization and diminished vascularization. In contrast the lungs tissues corresponding to the canalicular stage (*n* = 6) of development exhibited more organized and expanded parenchyma, indicating progressive alveolar differentiation, in association with increased vascularization. The younger lungs in pseudoglandular stage exhibited higher proportion of mesenchyme-rich regions indicating ongoing mesenchymal remodeling, while the more mature lungs corresponding to canalicular stage of development showed increased immune cell presence, indicating potential immune activity or ongoing tissue remodeling. Notably, the megakaryocyte infiltrated parenchyma and pulmonary vessels clusters expanded in later stages, consistent with ongoing immune and vascular maturation. These spatial annotations underscore the progressive regionalization of tissue architecture during human lung development (Figure S6).

**Fig. 3:**
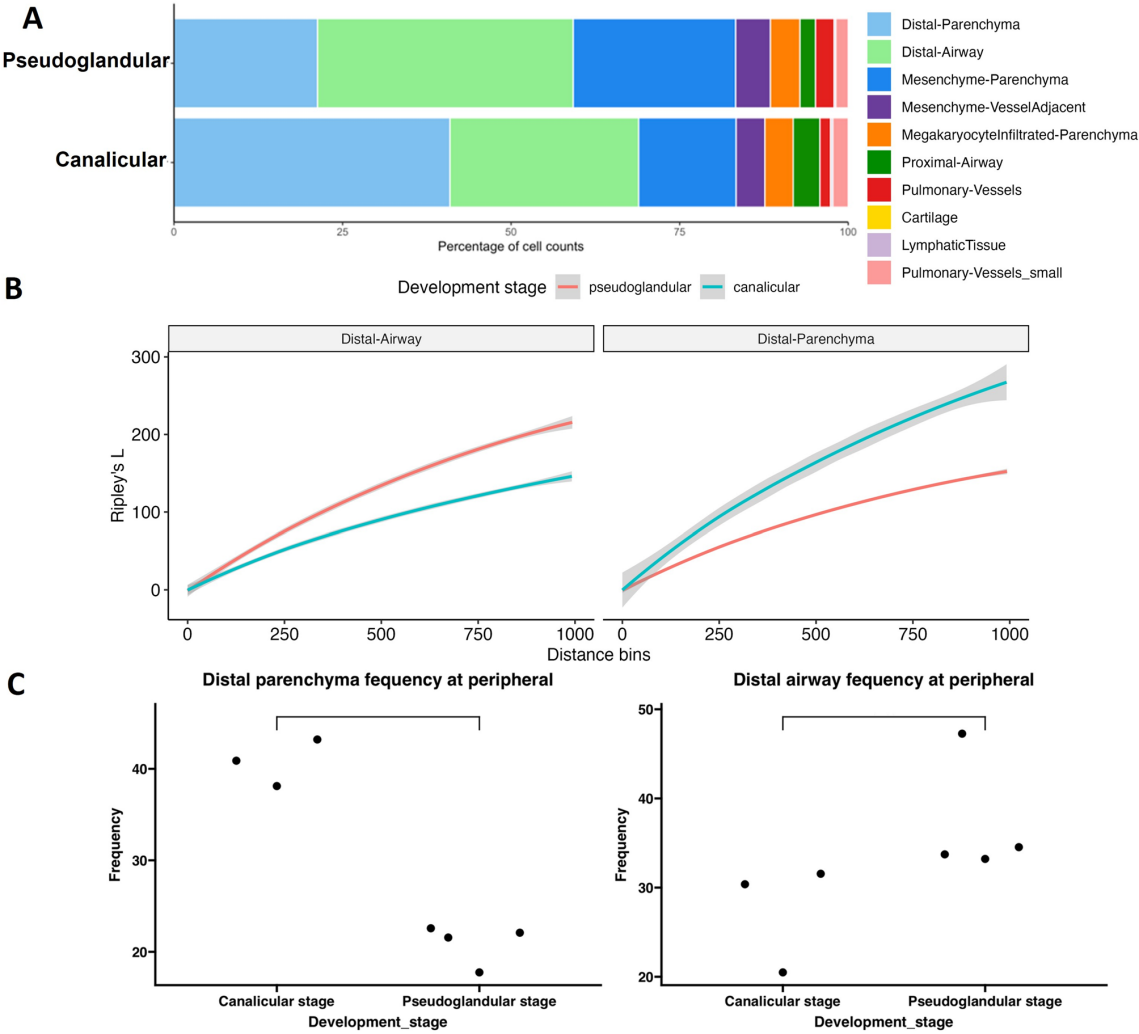
Spatial shift in human prenatal lungs with maturation. (**A**) Differences were observed in proportion of different spot types as the lungs develop from pseudoglandular to canalicular stage. Shown are the proportion of spots in prenatal lung tissue samples across pseudoglandular and canalicular stages. (**B**) Shift in Cellular Lineages in Peripheral Spots. Shown here are clusters of spots on tissue sections from lungs at 15 weeks of gestation (pseudoglandular stage) and at 19 weeks of gestation (canalicular stage). (**C**) Shift in Frequency of lineages in Peripheral Spots. Shown here are frequencies of alveolar and mesenchymal parenchymatous spots in lung tissue samples belonging to pseudoglandular and canalicular stages. Each dot indicates the frequency of the particular cell type in individual samples.

An examination of cluster markers and cellular annotations indicated an increase in the proportion of spots containing epithelial cells as the lung progresses through the stages of development (Fig. 3A). We observed that, during the transition from pseudoglandular to early canalicular stage, peripheral distal spots transition into airways, while more centralized spots retain mesenchymal characteristics. In peripheral regions (Figure S7), we observed higher frequency of spots annotated as of mesenchymal origin in pseudoglandular stage, compared to the non-peripheral spots (Fig. 3B). Proportional differences in the distribution of cells across the different clusters of prenatal lung spots over time indicated a statistically significant upward shift in the alveolar (epithelial) parenchyma and downward shift the distal (mesenchymal) parenchyma during the transition from pseudoglandular to canalicular stages (Fig. 3C).

Dispersion analysis using Ripley’s L statistic demonstrated developmental spatial structuring of the prenatal lung at different scales, with distal regions showing strong organization (Figure S4). While both distal parenchyma and distal airway spots showed the highest degree of clustering across all samples, the mesenchymal and immune-infiltrated parenchyma regions also display clustering, though at lower intensities, which suggests strong spatial organization within these regions. The canalicular samples exhibit stronger clustering in distal parenchyma and distal airway compared to others. Canalicular samples showed higher Ripley’s L values at local (smaller bin number) and whole sample (larger bin number) scales for distal parenchyma and distal airway spots compared to the pseudoglandular samples, suggesting increasing spatial organization and compartmentalization as lung development progresses (Fig. 3B). The increasing separation of distinct clustering curves in canalicular samples reflects progressive tissue differentiation and compartmentalization during development. In the pseudoglandular samples, several spot types showed similar clustering trends, with less distinct separation between mesenchyme, airway, and vascular components, while in the canalicular samples, distinct clustering patterns emerge, particularly in distal parenchyma and pulmonary vessels, indicating spatial specialization of these regions over time. Also, in the pseudoglandular lungs, mesenchyme-parenchyma and immune-infiltrated parenchyma show moderate clustering, suggesting early organization of stromal and immune niches, while in canalicular lungs these regions exhibit relatively lower clustering compared to distal structures, indicating a shift towards epithelial and vascular compartmentalization. These findings highlight the emergence of spatial tissue architecture in the developing human lung and demonstrate the utility of spatial transcriptomics for quantifying developmental patterning. The specific geometric features of Visium technology mitigate the influence of cell density on the Ripley’s L function measurement used in this study. Visium spots have a 55-micron diameter and are arrayed with a 100-micron center-to-center spacing, creating a 45-micron interstitial gap between the circular capture area^29,30^. The Ripley’s L function analysis here only assesses the differential distribution of pre-defined categorical spot types. If the analysis were instead applied to the distribution of deconvoluted cell types, variations in cell density would introduce a major confounding factor.

### *Transcriptomic changes across developmental stages*

**Fig. 4:**
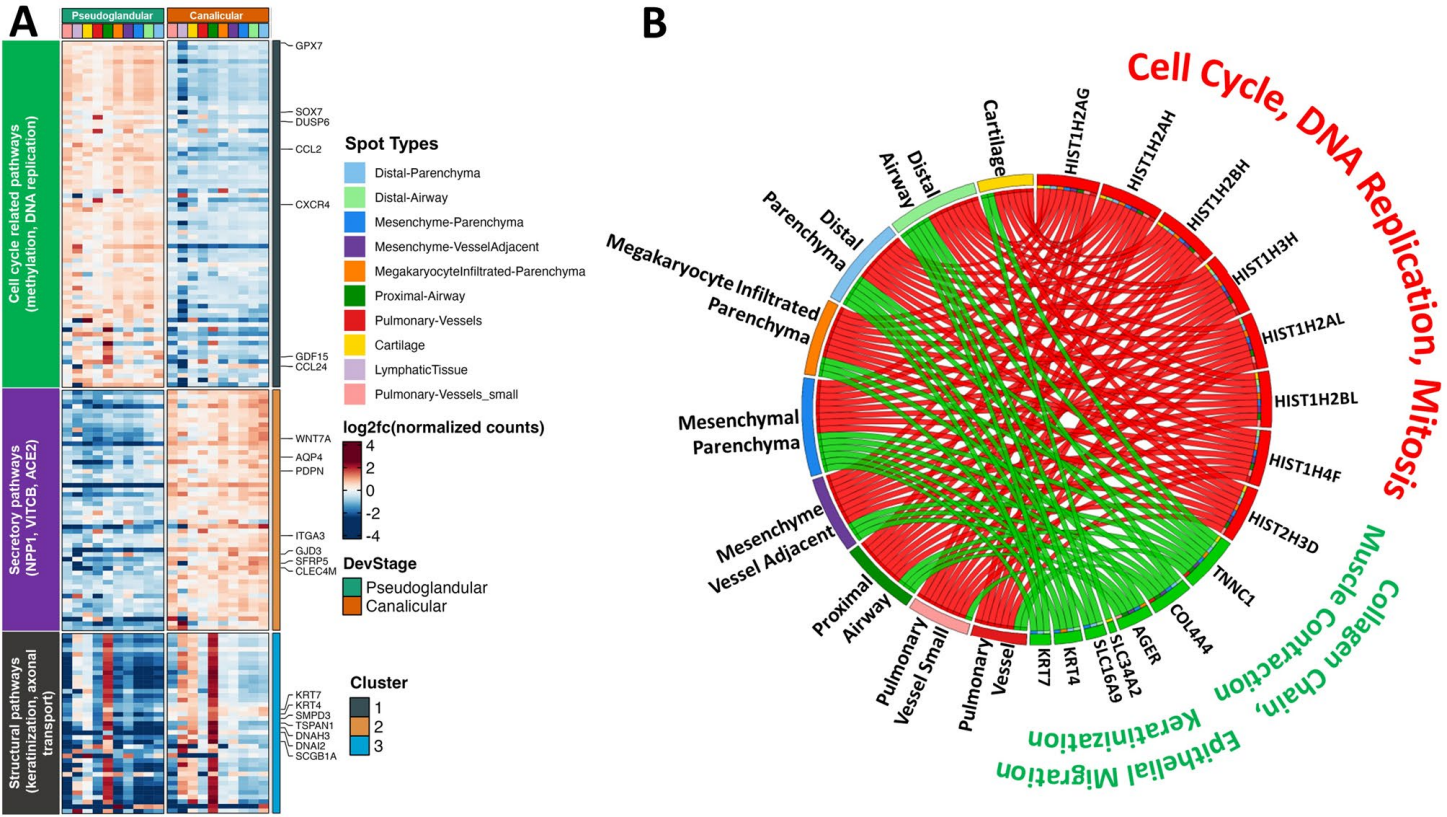
Transcriptomic changes in human prenatal lungs with maturation (**A**) Differential gene expression. Shown here is a heatmap of 166 genes that are differentially expressed between samples from pseudoglandular and canalicular stages. Each row represents a gene, and a column represents a spot type (color coded according to the legend). Plotted are log normalized fold change values with red indicating upregulation and blue is downregulation. (**B**). Pathway analysis using the genes differentially expressed between the pseudoglandular and canalicular stages. Shown here is a circus plot with select genes on the right and spot clusters on the left. Directionality of changes in genes and associated pathways in pseudoglandular stage compared to canalicular stage indicated by the color of the connecting links and pathway titles, where upregulation is indicated by red, and downregulation by green.Transcriptomic changes in human prenatal lungs with maturationTranscriptomic changes in human prenatal lungs with maturation

To characterize stage-specific transcriptional dynamics across distinct anatomical regions of the developing lung, differential gene expression assessment was performed by pseudobulk analysis (by DESeq2^31^ at FDR < 0.05) across all spot clusters, between the pseudoglandular (*n* = 6) and canalicular (*n* = 6) stages. Differential expression assessment identified 166 (114 upregulated and 52 downregulated) genes as significantly different in pseudoglandular samples, compared to those in the canalicular stage (Fig. 4A). Genes upregulated during the pseudoglandular stage included canonical histone and mitotic regulators such as *HIST1H2AG*, *HIST1H2AH*, and *TOP2A*, and were predominantly associated with cell cycle progression, mitosis, and DNA replication, consistent with high proliferative activity during early lung development. In contrast, the canalicular stage was marked by induction of genes involved in epithelial maturation (e.g., *KRT7*, *SFTPD*, *SCGB1A1*), secretory and ion transport pathways (*AQP4*, *PDPN*, *SLC6A9*), and structural remodeling (e.g., *COL4A4*, *TNNC1*), reflecting the onset of functional differentiation.

A survey of the differentially expressed genes within each spot type and the extent of overlap across regions, revealed both shared and region-specific transcriptional paradigms (Figure S8). Notably, several of these were shared among multiple mesenchymal and epithelial compartments, including the distal parenchyma, distal airway, and mesenchyme adjacent to vessels, highlighting conserved developmental transitions during lung morphogenesis. In fact, the largest intersect set of 24 differentially expressed genes is shared across multiple mesenchymal and epithelial compartments, suggesting common stage-dependent transcriptional changes in these regions. Conversely, subsets of DEGs unique to specific spot types, such as lymph node or pulmonary vessel, underscore spatially restricted regulatory programs that may reflect niche-specific cellular differentiation or microenvironmental cues.

Pathway analysis of the pseudoglandular-enriched genes showed strong association with pathways related to cell cycle and DNA synthesis, which aligns with the rapid cellular proliferation and tissue growth characteristic of the pseudoglandular stage, while canalicular-enriched genes (green links) were linked to secretory molecules (NPP1, VITCB, ACE2), keratinization, epithelial migration, and extracellular matrix organization (Fig. 4B). These data underscore a developmental shift from proliferative expansion to functional differentiation and tissue remodeling as the lung transitions into the canalicular stage.

When compared within individual spot clusters, we observed distinct patterns of pathway regulation across different spatial domains during the transition from pseudoglandular to canalicular stages of development. DNA methylation and chromatin modification pathways also show a trend towards upregulation in the pseudoglandular stage in several cell types, indicating active epigenetic remodeling during this phase. In addition, transcription regulation and nucleosome pathways show upregulation in the pseudoglandular stage in distal airway and distal parenchyma spot, suggesting active transcriptional and chromatin organization during this period of rapid differentiation. Immune system related pathways were predominantly upregulated in the canalicular stage within multiple different spot types, namely cartilage, proximal airway, mesenchyme vascular adjacent, and distal airway, indicating an increasingly important role for immune system in the maturation of these structures. Interestingly, surfactant metabolism pathway is specifically upregulated in the canalicular stage within the distal parenchyma spots, an observation consistent with the initiation of surfactant production in the developing alveolar epithelium during this stage. Integrin binding pathway was upregulated in the canalicular stage in megakaryocyte infiltrated parenchyma and vessel adjacent mesenchyme spots, reflecting potential changes in cell-matrix interactions. Overall, this comparative pathway analysis highlights the distinct temporal regulation of key biological processes within different spatial compartments of the developing lung, providing insights into the cellular mechanisms underlying the transition from the pseudoglandular to the canalicular stage.

## **Discussion**

In this study, we applied spatial transcriptomics to human prenatal lung tissue to capture region-specific transcriptional paradigms during early lung development. Integration of spatially resolved gene expression profiles, with cell type annotations derived from single cell sequencing, revealed both conserved and spatially restricted gene expression patterns across the pseudoglandular and canalicular stages, offering new insight into how structural and cellular complexity emerges during human lung morphogenesis.

We identified ten distinct transcriptional niches across epithelial, mesenchymal, vascular, and immune compartments. Our data has captured well-characterized developmental hallmarks, including the progressive expansion of alveolar epithelium and the decline of mesenchymal tissue as gestation advances. It has previously been reported that during the embryonic stage of lung development, the mesenchymal cells surround the epithelial cells, and signal the initiation of branching morphogenesis, while at the pseudoglandular stage, the epithelial cells initiate the expression of airway lineage markers^32,33^.

Our data has captured well-characterized developmental hallmarks, including the progressive expansion of alveolar epithelium and the decline of mesenchymal tissue as gestation advances. The application of spatial transcriptomics through the 10X Visium platform has enabled precise spatial localization of the developmental transitions. We observed a shift from mesenchymal to epithelial spot types during the pseudoglandular-to-canalicular transition, particularly within the peripheral regions of the developing lung. Proportional differences in the distribution of cells across the different clusters of prenatal lung spots over time indicated an upward shift in the alveolar (epithelial) parenchyma and downward shift the distal (mesenchymal) parenchyma during the transition from pseudoglandular to canalicular stages. This observation is consistent with our previous report of increase in proportion of epithelial cells in normal prenatal lung at 18 weeks of gestation compared to one at 11 weeks^14^. Distal-airway cells act as intermediaries between the large airway epithelium and the distal gas exchange region, retaining airway lineage genes while housing developing progenitors. Distal parenchyma cells are committed toward the alveolar fate, with increasing specialization for surfactant metabolism and alveolar structure as gestation advances. These distinctions are reflected in both cellular composition and spatial/temporal gene expression dynamics during human lung organogenesis.

Our findings broadly confirm and complement major insights from the recent spatial transcriptomics studies^21,22^. He et al. created a comprehensive atlas of human fetal lung cellular diversity through integrative single-cell and spatial analyses, uncovering over 140 cell states, identifying novel epithelial and mesenchymal progenitors, and mapping their distribution along proximal-distal and niche-specific gradients across development. To ensure robust and accurate cell-type annotation across samples with varying regional representation, we leveraged the publicly available single-cell RNA-seq reference dataset from He et al. (2022) for deconvolution of Visium spots in our study. The He et al. dataset provides comprehensive coverage of fetal lung cell types spanning 10–22 weeks of gestation, which encompasses but also extends beyond the more focused interval of 13–20 weeks gestation interrogated in our work. By focusing our analyses on this narrower developmental window and integrating well-validated external reference data, we minimized heterogeneity related to sampling location and developmental timing. Although some samples captured predominantly peripheral or region-specific tissue sections (as illustrated in Fig. S6), the combination of high-resolution single-cell references and targeted gestational age selection ensured the biological consistency and interpretability of our spatial transcriptomic comparisons. Similarly, Quach et al. highlighted the temporal emergence of new epithelial progenitors and detailed their transition into mature cell types, while resolving dynamic cell–cell signaling networks and spatial interactions that shape lung morphogenesis. Building on these seminal works, our study extends this framework by including samples between 13 and 20 weeks post-conception encompassing the transition from the pseudoglandular to canalicular stages of development, enabling direct spatial comparison of normal and abnormal developmental trajectories. By applying state-of-the-art deconvolution and integration methodologies, we confirm key stage-specific changes in cellular composition and spatial organization and uniquely characterize how genetic perturbation may (or may not) influence these processes in situ. Thus, our results not only validate core principles of lung patterning and niche specifications defined by the two aforementioned studies^21,22^, but also broaden their context to encompass developmental aberrations relevant to human disease.

The increasing clustering of distal parenchymal and distal airway regions in the canalicular lungs, as measured by Ripley’s L statistic, indicates increasing spatial organization and compartmentalization as the lung develops. Classical experiments and more recent molecular lineage analyses demonstrate that reciprocal signaling interactions between endoderm-derived epithelial cells and mesenchyme derived from the splanchnic mesoderm are critical for lung patterning, branching morphogenesis, and cell fate specification. As lung development proceeds from the pseudoglandular to the canalicular and saccular stages, these compartments become increasingly distinct, with the epithelial domain elaborating into specialized airway and alveolar lineages, and the mesenchyme giving rise to structurally and functionally diverse cell types. Our observations suggest that the process of lung maturation is not only marked by changes in cellular composition, but also by enhancement of regional specificity and structural refinement (Figure S4). This is consistent with prior imaging and lineage-tracing studies that have shown progressive spatial delineation of epithelial and mesenchymal compartments during prenatal lung development^4,34^.

At the transcriptomic level, differential expression analysis revealed a developmental shift from proliferative to differentiated processes. Pseudoglandular lung samples were enriched for genes involved in cell cycle and chromatin organization, consistent with high proliferative activity. Similar transcriptional programs have been observed in bulk and single-cell RNA-seq studies of human and mouse lungs, where early developmental stages show enrichment in proliferative progenitors and chromatin modifiers^35,36^. In contrast, canalicular lungs exhibited upregulation of pathways related to epithelial differentiation, surfactant metabolism, secretory function, and extracellular matrix remodeling, which are hallmarks of functional maturation. Notably, surfactant-associated genes such as *SFTPD* and epithelial markers like *KRT7* were specifically enriched in distal parenchymal regions, reflecting their importance in alveolar epithelial differentiation. While direct in situ visualization (using IHC or ISH) of selected mesenchymal and immune markers at each developmental stage would further support the spatial gene expression findings, such experiments are planned for future work or in follow-up studies. In-situ approaches indicating the region-specific expression of surfactant proteins and differentiation markers described in prior studies^37,38^ have already highlighted the feasibility and relevance of this validation.

Pathway analyses of the differentially expressed genes across the different spot types have demonstrated that the developmental transitions do not occur uniformly across the different spot types, but there are spot-specific regulatory programs involved in the process. While DNA methylation and nucleosome assembly pathways were upregulated in distal epithelium and airways during the pseudoglandular stage, immune-related pathways had greater prominence in the canalicular stage in the mesenchymal and airway regions. These findings align with previous studies highlighting epigenetic priming of epithelial precursors^39^ and a temporal increase in immune signaling components during late canalicular to saccular stages^36,40^. These observations demonstrate the interaction between differentiation and microenvironmental signaling during the compartmentalization of the lung.

Most importantly, this study adds spatial context to established developmental paradigms. Our findings support the notion that, at the tissue level, the mesenchymal-to-epithelial transitions during lung development occur in a peripheral-to-central gradient and are tightly coupled with vascular and immune maturation. This gradient has been previously described through clonal and spatial analyses showing peripheral progenitor expansion and central lineage diversification^34,41^. These patterns highlight how coordinated lineage specification and regional structuring contribute to lung readiness for air breathing at birth.

While this study provides valuable insights into the spatial transcriptomic landscape of prenatal lung development, some limitations should be considered. While the sample size (*n* = 12) is modest, our dataset covers samples across a critical gestational window, which incorporates the transition from pseudoglandular to canalicular stage of development. Data consistency across samples supports the robustness of our core findings. Larger sample numbers in future studies may enable detection of subtler stage- or genotype-specific effects but are not expected to change our main conclusions. While our study includes four Trisomy 21 cases, our analysis did not show any systematic clustering or differences in distribution of spots associated with this diagnosis (Figure S10 and Table S1). In its current structure, the dataset is severely underpowered to detect differences between normal and trisomy 21lungs within each stage due to low number of diseased samples, there was no difference in distribution of spot type frequencies across the two conditions (*p* = 0.962) using permutation test^42^. Furthermore, pseudobulk analysis for differential expression for Trisomy 21 identified only 32 genes, none of which were among the 166 genes differentially expressed between the early lung development stages. Additionally, the dataset does not extend into later canalicular or saccular stages when alveolarization and surfactant production intensify. Inclusion of these stages in future studies will be critical to fully map lung maturation. Given subtle differences in spot distributions observed, analysis with separate stratification for T21 and control samples may be more informative in future studies.

Despite these limitations, this work provides a valuable spatial framework for interpreting prenatal lung development. This study not only confirms the cellular transitions observed in previous scRNA-seq atlases^13–15^, also adds spatial context to these findings, and uniquely expands on prior spatial transcriptomics reports^21,22^ by incorporating advanced spatial deconvolution. By integrating spatial transcriptomics with single-cell references, we have established a topographic atlas of developmental paradigms across anatomical compartments. This atlas serves as a foundation for future studies of congenital lung disorders, including pulmonary hypoplasia and bronchopulmonary dysplasia, where deviations from normal spatial patterns may lead to underlying disease pathogenesis. Ultimately, these results advance our understanding of how structural, cellular, and molecular intimations converge to orchestrate human lung development in situ.

## **Methods**

Following informed consent, de-identified human prenatal samples were collected under IRB approval (The Lundquist Institute, 18CR-32223-01) and provided by The University of Washington Birth Defects Research Laboratory (BDRL) and all the methods in the study were performed in accordance with relevant guidelines and regulations. Since this study utilized previously collected deidentified samples with no access to identifiable information, it falls under exemption 4 of 45 CFR part 46 or the Basic HHS Policy for Protection of Human Research Subjects (45 CFR 46.101(b)(4)) requirements. The demographic information provided was limited to gestational age, known genetic conditions, or gestational complications.

### ***Sample collection ***

Spatial transcriptomic profiles were generated using lung tissue sections obtained from twelve prenatal human lung samples ranging in gestational age from 13 to 20 weeks, of which four were diagnosed with Trisomy 21, with defined hypoplasia. Lungs were fixed in 4% PFA for immunohistochemical analyses as discussed previously^15^. Lung tissue sections were assessed for quality using DV200 criteria, and those with DV200 values greater than 50% were selected for spatial transcriptomics. 5-micron tissue sections from individual samples were placed onto Visium Gene Expression Slides (10X Genomics), stained with H&E using Visium chemistry (v1) and scanned, in two batches. Subsequently, the tissue samples were permeabilized to release ligated probe pairs from the cells, that are bound to the spatially barcoded oligonucleotides present on the spots. The resulting barcoded libraries were sequenced on a NovaSeq 6000 sequencer (Illumina) and aligned to GRCh38-2020-A and quantified using the standard Spaceranger workflow (v2.0.1).

### Background correction, filtering, tissue edge labeling, and normalization

In the raw Visium images color unmixing was performed followed by eosin staining normalization using methods described previously^43,44^ targeted to remove and correct unwanted artifacts and uneven staining between samples. Briefly, the RGB values of the high-resolution eosin staining image (2000 × 2000 pixel^2^) generated by the 10x Spaceranger pipeline were converted to the optical density value. The transparent pixels were filtered out and the largest eigen vector was selected as the eosin signal, which was subsequently normalized against the reference pixel value using QuPath^45,46^. The normalized eosin staining images were subsequently converted to grey scale and quantified using Squidpy^47^, and the OD value of the 90th quantile of each 10x spot was used to filtered out tissue-sparse spots that was missed by 10x default algorithm. The spots adjacent to the background spots were defined as tissue edge spots.

The resulting count matrixes were then filtered again to remove any spot that has an RNA molecule count smaller than 200. The filtered molecular count data of each sample was individually modeled and normalized using the regularized variance stabilization (vst) method^48^ as implemented in Seurat^49^. To robustly assess global spatiotemporal changes in gene expression during development, we employed established batch effect correction and data integration pipelines (Harmony, SAM) that preserve true biological variance while minimizing technical confounders, ensuring that observed developmental trends reflect genuine spatiotemporal processes rather than sample-specific biases. Dimensional reduction, sample integration and Uniform Manifold Approximation Projection (UMAP) were performed using the Self Assembling Manifold (SAM)^50^ and Harmony^51^ algorithms as implemented in Seurat^25–27^.

### Unsupervised clustering and spot annotation

The top 150 principal components were used to calculate stabilized nearest neighbor (snn) score and generate clusters of spots using the smart local moving (SLM) method. The individual cluster markers were determined using the Wilcoxon Rank Sum test in the Seurat FindAllMarkers() function with default parameters. The spot clusters were annotated markers were run through multiple resources of ToppCellAtlas including, A Cellular Census of Human Lungs (Vieira Braga et al.), A spatial multi-omics atlas of the human lung reveals a novel immune cell survival niche, Human Fetal Lung in Development Atlas, Human Lung Cell Atlas (HLCA), Human embryonic and fetal stage lung cell atlas, The Integrated Human Lung Cell Atlas, and Tissue Stability Cell Atlas - Lung Cells^19,21,23,24^ collections of ToppFun^25,26^, and PanglaoDB^27^ in order to generate cluster annotations.

### Dispersion statistics

To compare the spatial dependence (spot clustering or spot dispersion) of different spot types between samples at Pseudoglandular and Canalicular stages, the variance-stabilizing transformed centroid distribution score (Ripley’s L) was calculated using the squidpy.gr.ripley() function in Squidpy^47^ for each sample, which provides a shape robust description of the deviations from spatial homogeneity. The sample level measurements were then reorganized by different spot type and subsequently aggregated using the local polynomial regression fitting into pseudoglandular and canalicular stages.

### Deconvolution

In order to gain a better insight into the cellular heterogeneity of individual spots, deconvolution was performed. We used Robust Cell Type Decomposition (RCTD) algorithm^28^ to deconvolute spatially resolved transcriptomic spots by leveraging single-cell RNA sequencing (scRNA-seq) data generated from prenatal human lung tissues^21^ as a reference model that captures the gene expression profiles of different cell types and states within a tissue with high granularity. The RCTD algorithm uses a probabilistic framework to assign proportions of cell types or states to each spatial spot, enabling precise dissection of the cellular composition within spatially heterogeneous regions. This method ensures accurate mapping of cellular distributions across regions of interest and allows for quantitative comparisons of cellular heterogeneity and tissue organization between normal and hypoplastic lungs.

### Defining tissue border and peripheral region

The tissue edge was determined during the initial quality control process. The tissue border spots were manually selected from the tissue edge spots that resemble the pleural surface. The radio distance (pixel) of each tissue spot to the nearest border spots were subsequently measured using the semla package^52^. We defined the peripheral region as the tissue spots that have radio distance not greater than approximately 500 microns (150 pixels) from the border spots, which was calculated from the mean spot and fiducial diameter in full resolution from each sample. The frequency of the distal parenchyma spot in the peripheral region and the non- peripheral region were compared using student test.

## Differential gene expression

The differential composition and gene expression assessments between the samples from pseudoglandular (gestational age of 16 weeks or less) and canalicular (gestational age of over 16 weeks) stages were performed using the sccomp^51^ and DESeq2^31^ packages respectively, incorporating for sex differences as a co-variate. The biological sex of each sample was determined by a male gene signature consisted of RPS4Y1, UTY, KDM5D, DDX3Y, USP9Y, VCX, VCY. The signature was calculated using the UCell algorithm^53^. Pathway analysis using the differentially expressed genes was performed using ToppGene tool of ToppFun^25,26^.

### Statistical analysis

No statistical method was used to predetermine the sample size. Frequencies were calculated in R, and for frequency comparison across stages and condition, the Wilcoxon ranked sum test and permutation test were used respectively.

**Table 1:** Subject demographics prenatal lung samples (*n* = 12) were used for Spatial transcriptomics using 10X Visium. Shown here are the demographic information for individual samples including gestational age, sex, histological stage, and tissue quality information.

Sample name	Gestational age (weeks)	Sex	No of spots	DV200	Pathology	Histological stage
*13WeekA*	13	Female	838	< 50%	No lung disease	Pseudoglandular
*13WeekB*	13	Male	3792	50–70%	No lung disease	Pseudoglandular
*15WeekA*	15	Male	3190	> 70%	No lung disease	Pseudoglandular
*16WeekA*	16	Female	4411	> 70%	Trisomy 21	Pseudoglandular
*16WeekB*	16	Female	3271	> 70%	No lung disease	Pseudoglandular
*16WeekC*	16	Male	4198	> 70%	Trisomy 21	Pseudoglandular
*17WeekA*	17	Female	4251	50–70%	No lung disease	Canalicular
*17WeekB*	17	Female	4146	> 70%	Trisomy 21	Canalicular
*18WeekA*	18	Female	3130	< 50%	No lung disease	Canalicular
*18WeekB*	18	Female	3251	50–70%	No lung disease	Canalicular
*19WeekA*	19	Male	4558	> 70%	Trisomy 21	Canalicular
*20WeekA*	20	Male	4399	> 70%	No lung disease	Canalicular

## Supplementary Information

Below is the link to the electronic supplementary material.

Supplementary Material 1

## Data Availability

Raw and processed data associated with the study are available in Gene Expression Omnibus (GEO) and can be retrieved using accession number GSE310610.
