# Supplementary material for: Spatial transcriptomics of developing human lungs defines cellular phenotypes associated with age, lineage and location
Source: Sci Rep. 2026 Jan 3;16:4573. doi: 10.1038/s41598-025-34594-z (PMC12868815; doi:10.1038/s41598-025-34594-z)
Supplement: Supplementary file 1 — Supplementary Material 1 [file 41598_2025_34594_MOESM1_ESM.docx]

**Supplemental Information for**

**Spatial Transcriptomics of Developing Human Lungs Defines Cellular Phenotypes Associated with Age, Lineage and Location**

Yue Ren*^1^*, Soula Danopoulos*^2,5^*, Gail H. Deutsch*^3^*, Ian A. Glass*^4^*, Thomas J. Mariani*^1^*, Soumyaroop Bhattacharya*^1^*

Affiliations

*^1^Center for Children’s Health Research, Department of Pediatrics, School of Medicine, University of Rochester, Rochester, NY.*

*^2^Lundquist Institute for Biomedical Innovation at Harbor-UCLA Medical Center, Torrance, CA, USA*

*^3^Department of Laboratory Medicine and Pathology, University of Washington School of Medicine and Seattle Children’s Research Institute, Seattle, WA, USA*

*^4^Department of Pediatrics, University of Washington School of Medicine, Seattle, WA, USA*

*^5^Department of Pediatrics, David Geffen School of Medicine at UCLA, Los Angeles, CA, USA*

**Figure S1: Quality Control metrics:** Shown here are expression levels of individual spots (A) and genes (B) distributed by their sample. Each dot represents a spot (A) or a gene (B).


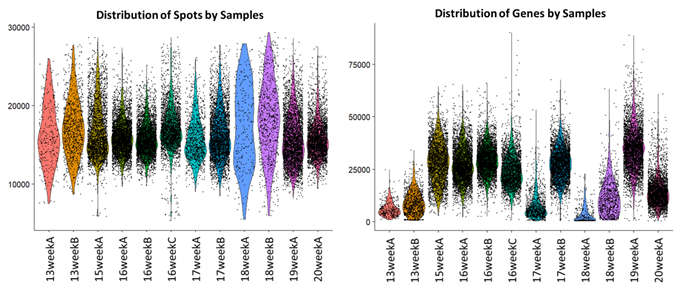


**Figure S2: Distribution of spots by Samples.** Uniform Manifold Approximation and Projection (UMAP) displays the 10 unique spot clusters. Shown here are spots from 12 samples ranging in gestational age of 13-20 weeks post conception, with each color representing an individual sample. Despite differences in gestational age, the UMAP reveals substantial overlap in transcriptional profiles across samples, suggesting conserved spatial gene expression programs during this window of lung development.


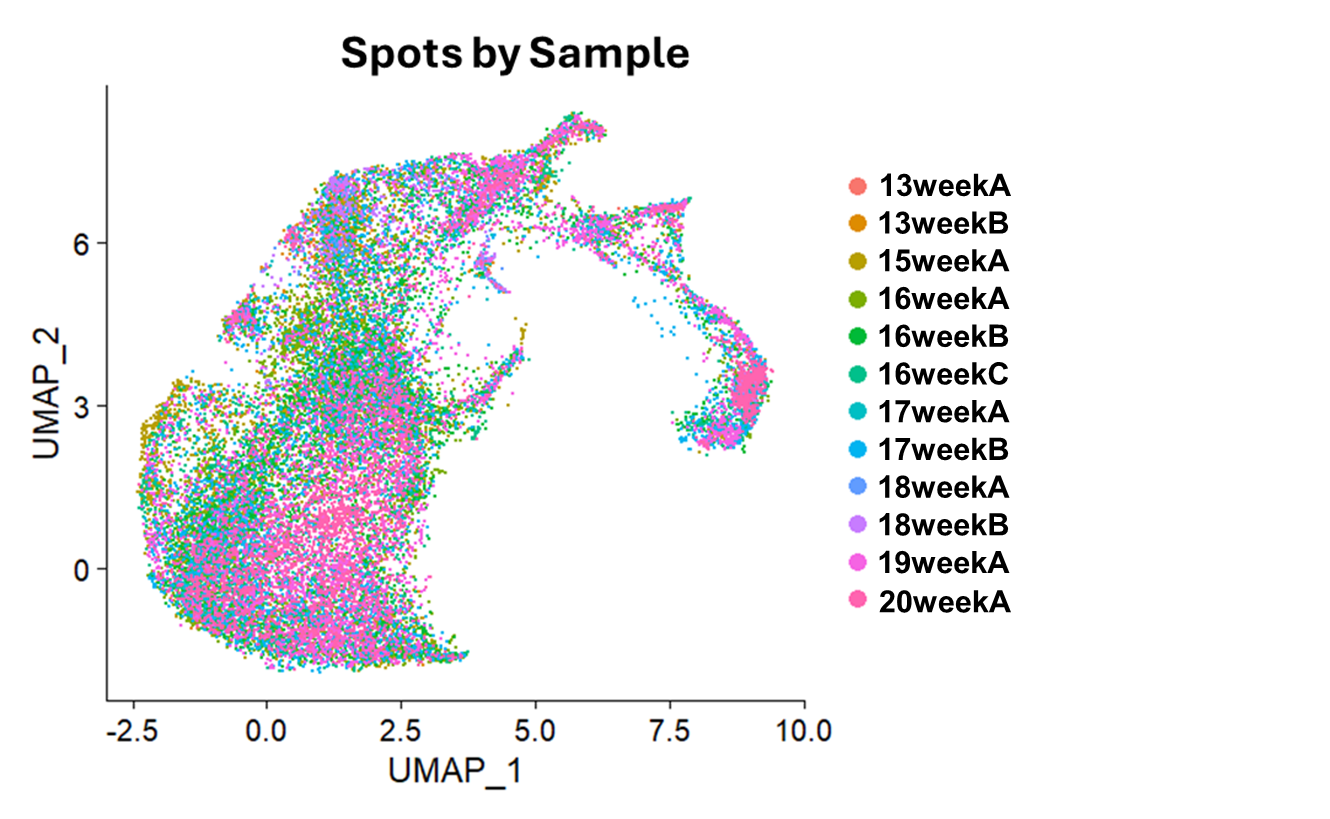


**Figure S3: Expression of some known cell type markers in the spots.**


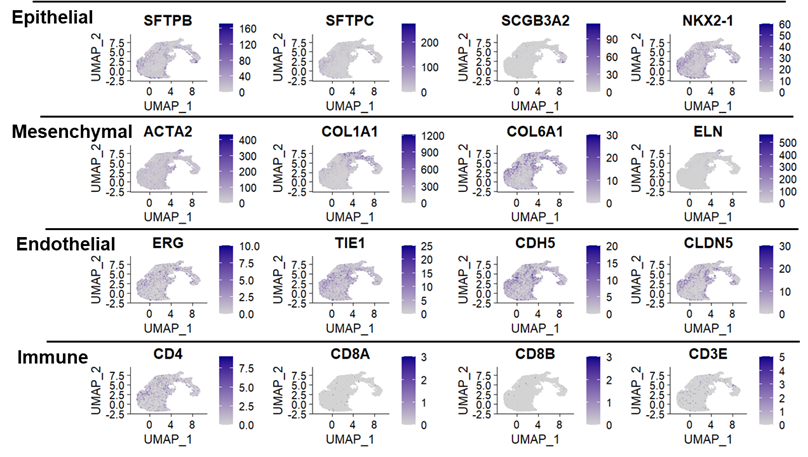


**Figure S4: Spatial Dispersion analysis to assess distribution of spot clusters across developmental stages.** Shown here are Ripley’s L curves for 7 prenatal lung samples profiled by Visium spatial transcriptomics. Each line represents the spatial dispersion of a specific spot type, with greater curve height indicating higher spatial clustering at increasing spatial distances (bins). Distal parenchyma (orange) and distal airway (blue) spot types consistently demonstrate higher spatial clustering compared to other tissue compartments, reflecting structured regional organization during lung development.


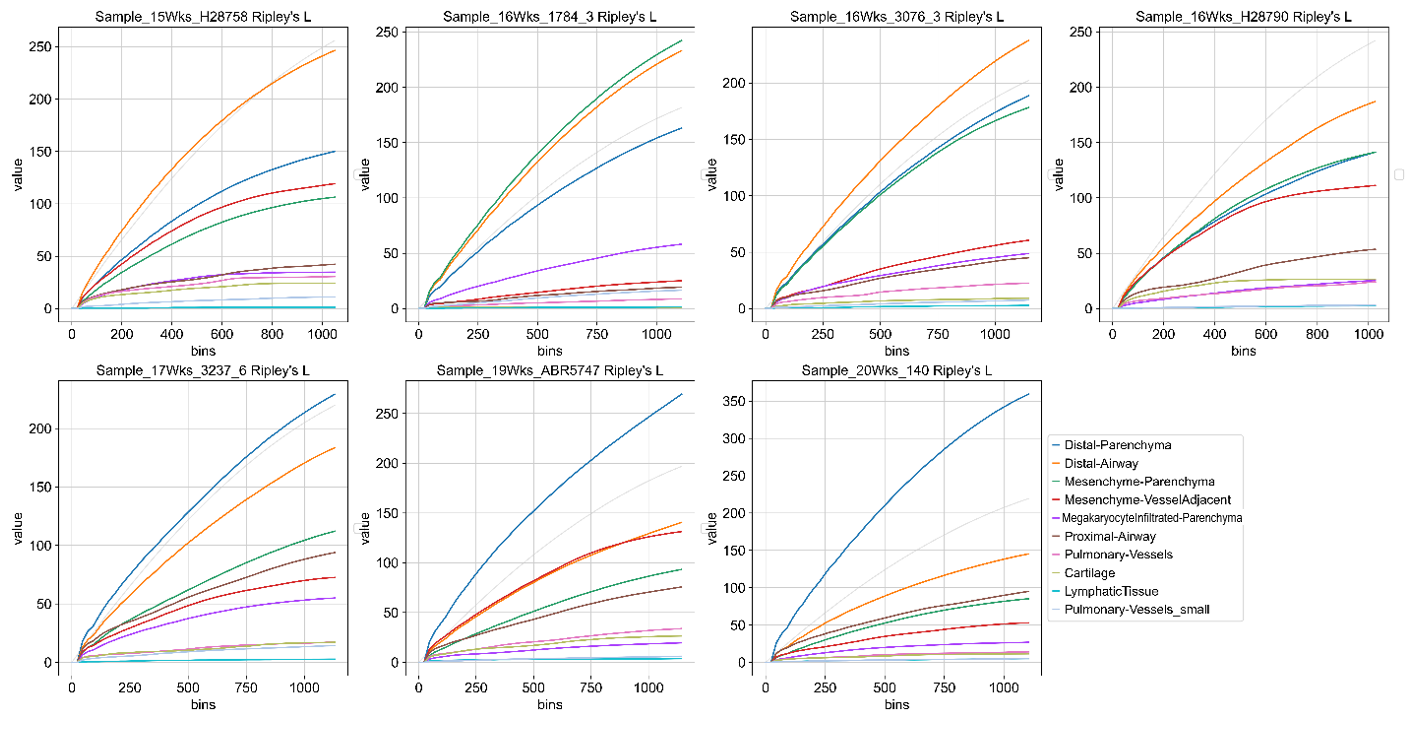


Figure S5: Proportion of cells within individual spot clusters.

Figure S6: Spatial distribution of spot clusters. Shown here are clusters of spots on tissue sections obtained from human prenatal lungs between 15-20 weeks of gestation post conception. Spots are colored by the cell type annotation presented in the legend.


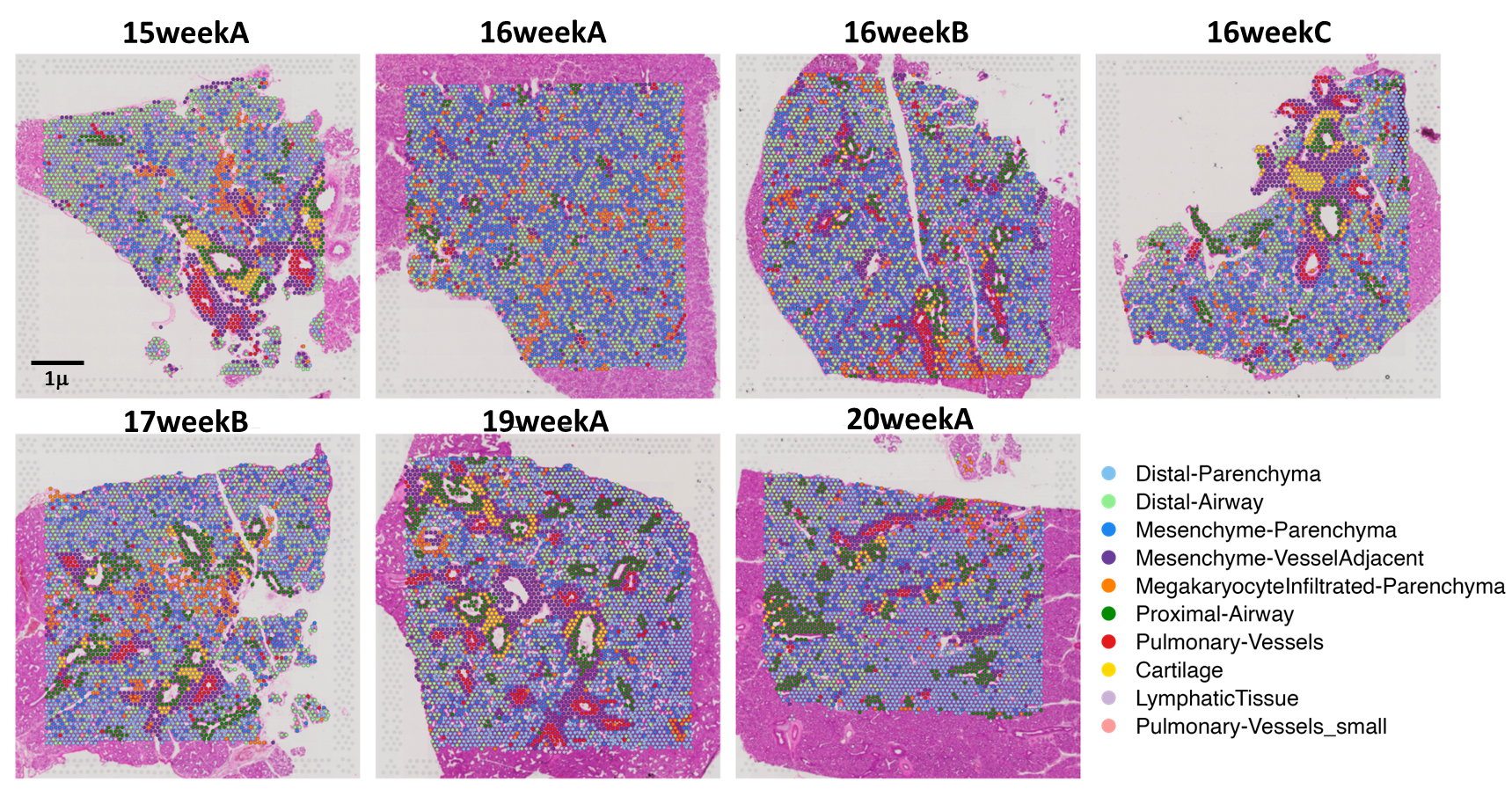


Figure S7: Peripheral regions in lung tissue sections. Peripheral regions in each of the individual lung tissue sections were defined as the region of 500 micron from the edges of the tissues. The distance was calculated based on the total resolution of the image.


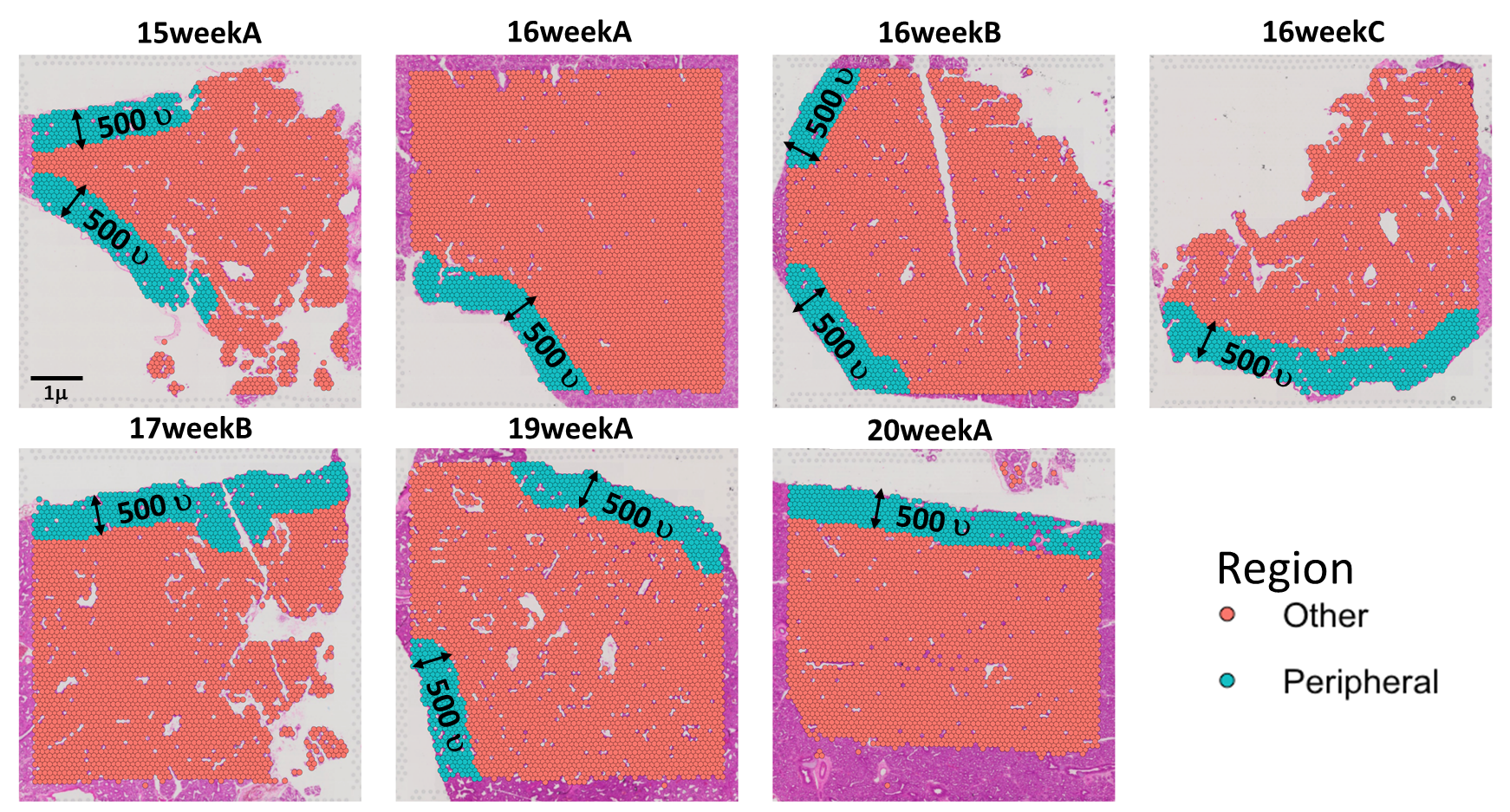


Figure S8: Distribution of differentially expressed genes across spot types. Shown here is an upset plot indicating the number and intersection of differentially expressed genes identified within each spot type in the prenatal lung tissue. Horizontal bars on the left represent the total number of DEGs within each individual spot type. Vertical bars indicate the size of each intersection, i.e., the number of genes shared among specific spot types, with connected dots below each bar showing the corresponding combination of spot types.


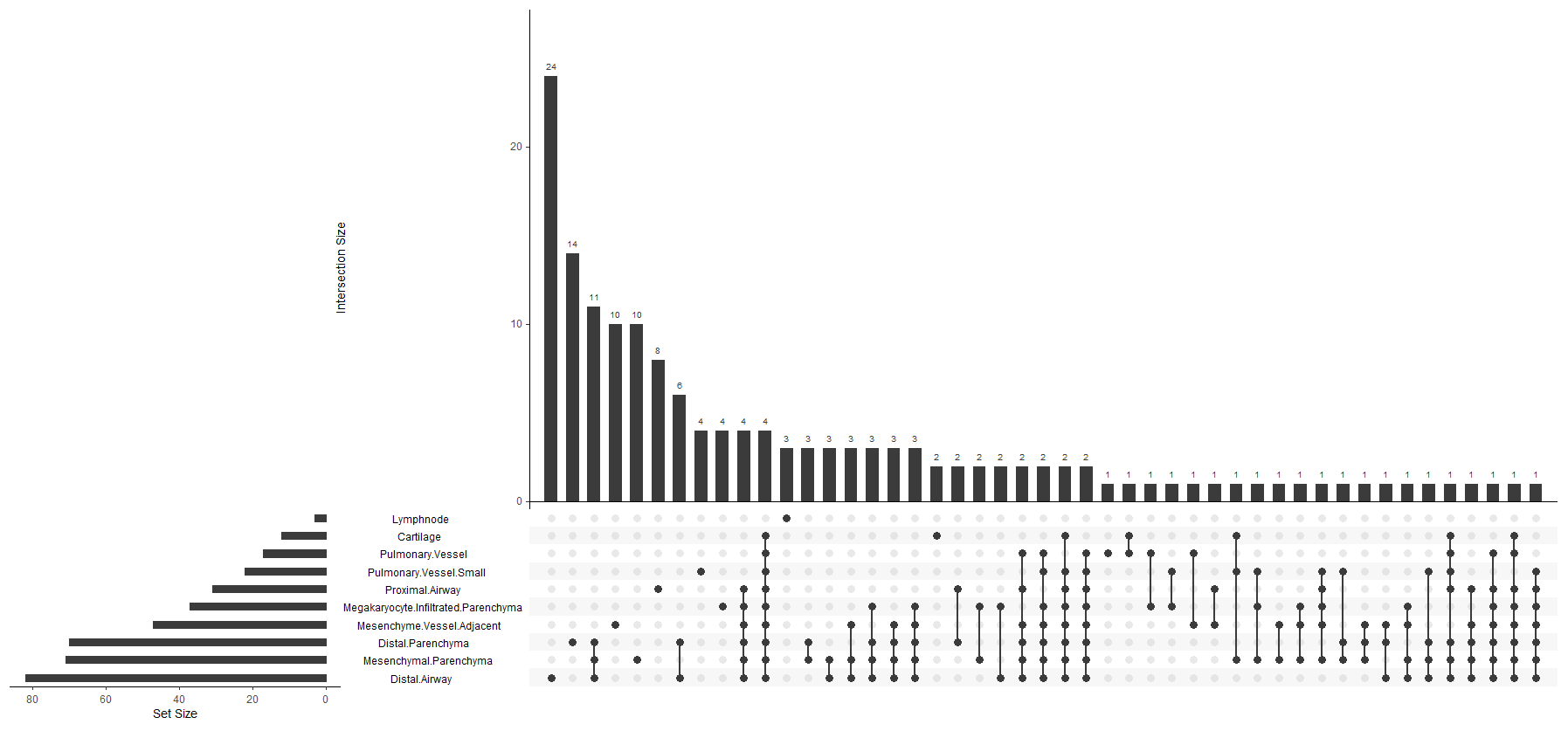


Figure S9: Pathways altered during shift from pseudoglandular to canalicular stage within individual spot clusters. Shown here is a summary heatmap visualizing the significantly enriched pathways (columns) across the different spatial spot clusters (rows). Red indicates pathways significantly upregulated in the pseudoglandular stage, while green indicates pathways significantly upregulated in the canalicular stage within a specific cell type.

|  | **Immune System** | **Innate Immune response** | **Neutrophil Degranulation** | **Complement Activation** | **Surfactant Metabolism** | **Integrin Binding** | **DNA Methylation** | **Cell Cycle** | **Chromatin Modification** | **Nucleosome** | **Transcription Regulation** | **DNA Replication** |
| --- | --- | --- | --- | --- | --- | --- | --- | --- | --- | --- | --- | --- |
| *Cartilage* |  |  |  |  |  |  |  |  |  |  |  |  |
| *Distal Airway* |  |  |  |  |  |  |  |  |  |  |  |  |
| *Distal Parenchyma* |  |  |  |  |  |  |  |  |  |  |  |  |
| *Megakaryocyte Infiltrated Parenchyma* |  |  |  |  |  |  |  |  |  |  |  |  |
| *Lymphatic Tissue* |  |  |  |  |  |  |  |  |  |  |  |  |
| *Mesenchyme Parenchyma* |  |  |  |  |  |  |  |  |  |  |  |  |
| *Mesenchyme Vessel Adjacent* |  |  |  |  |  |  |  |  |  |  |  |  |
| *Proximal Airway* |  |  |  |  |  |  |  |  |  |  |  |  |
| *Pulmonary Vessels* |  |  |  |  |  |  |  |  |  |  |  |  |
| *Pulmonary Vessels Small* |  |  |  |  |  |  |  |  |  |  |  |  |

Figure S10: Distribution of spots across clusters separated by disease condition. (A) Uniform Manifold Approximation and Projection (UMAP) displays the 10 unique spot clusters split by their diagnosis of Trisomy 21 (T21) and no lung disease. Shown here are spots from 12 samples ranging in gestational age of 13-20 weeks post conception, with each color representing a cluster of spots. (B) Uniform Manifold Approximation and Projection (UMAP) displays the 10 unique spot clusters split by their histological stage (pseudoglandular or canalicular). Shown here are spots from 12 samples ranging in gestational age of 13-20 weeks of gestation, with each color representing their diagnosis of Trisomy 21 (T21) and no lung disease.

A
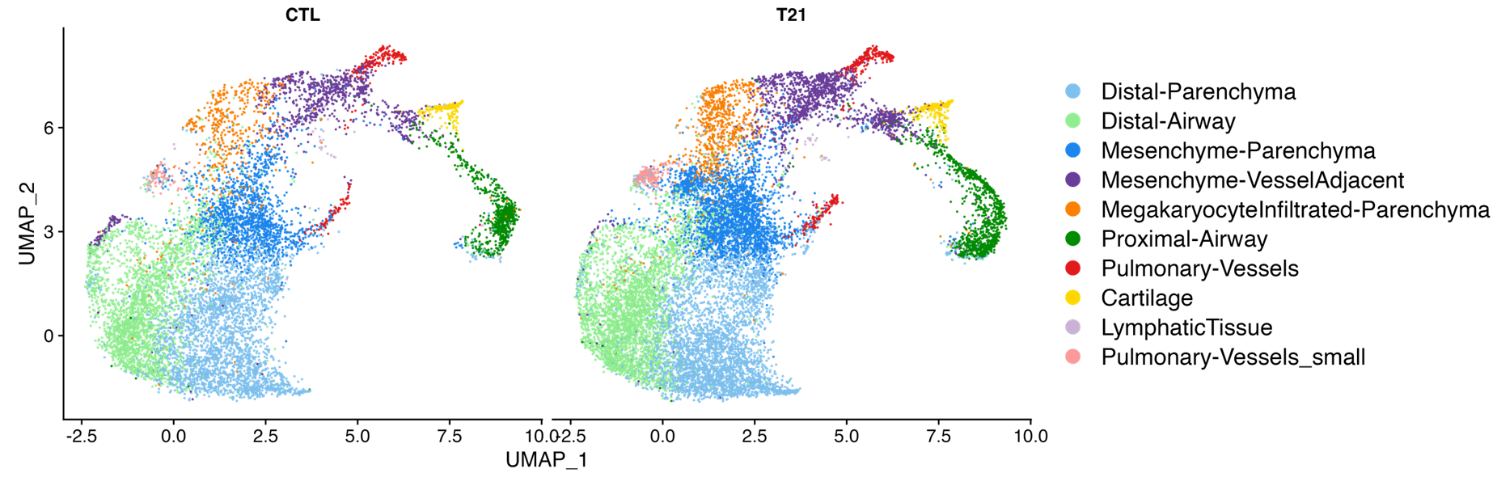


B
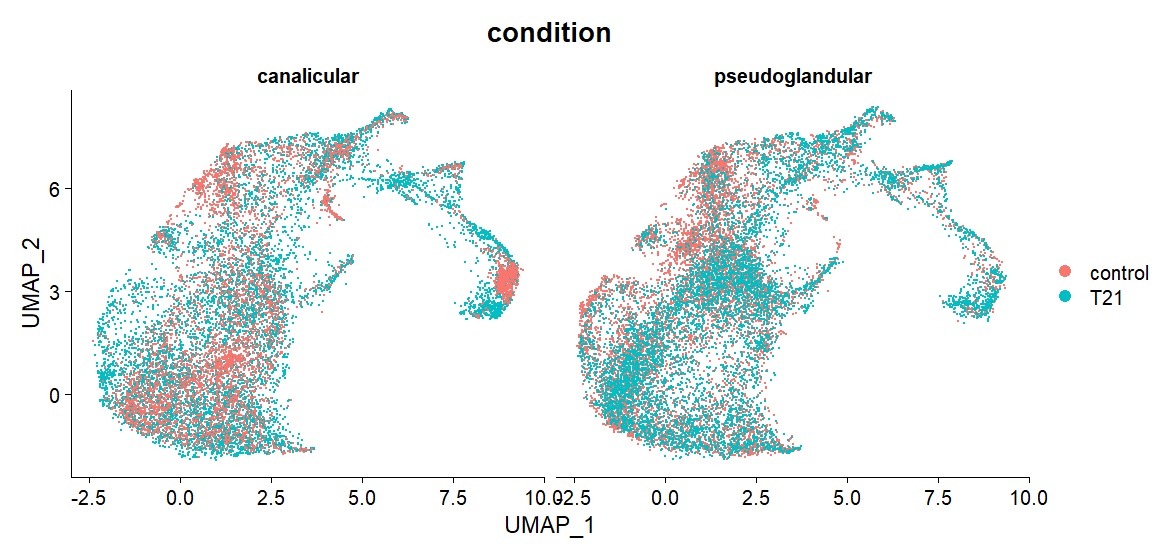


Table S1: Distribution of spots by samples

| **Spot Type Annotation** | **13 Weeks A** | **13 Weeks B** | **15 Weeks A** | **16 Weeks A (T21)** | **16 Weeks B** | **16 Weeks C (T21)** | **17 Weeks A** | **17 Weeks B (T21)** | **18 Weeks A** | **18 Weeks B** | **19 Weeks A (T21)** | **20 Weeks A** |
| --- | --- | --- | --- | --- | --- | --- | --- | --- | --- | --- | --- | --- |
| *Distal Parenchyma* | 3 | 31 | 559 | 923 | 950 | 612 | 138 | 1085 | 0 | 14 | 1432 | 1584 |
| *Distal Airway* | 37 | 115 | 961 | 1274 | 1181 | 789 | 329 | 841 | 7 | 56 | 719 | 641 |
| *Mesenchyme-Parenchyma* | 30 | 152 | 369 | 1315 | 849 | 564 | 126 | 511 | 12 | 74 | 465 | 365 |
| *Mesenchyme Vessel-Adjacent* | 46 | 21 | 429 | 135 | 294 | 421 | 45 | 293 | 20 | 147 | 619 | 220 |
| *Megakaryocyte Infiltrated-Parenchyma* | 151 | 505 | 119 | 317 | 254 | 103 | 198 | 227 | 101 | 376 | 104 | 114 |
| *Proximal Airway* | 0 | 0 | 155 | 101 | 210 | 210 | 98 | 391 | 13 | 60 | 370 | 423 |
| *Pulmonary Vessels* | 2 | 2 | 113 | 53 | 109 | 103 | 17 | 74 | 0 | 13 | 170 | 58 |
| *Cartilage* | 8 | 0 | 82 | 6 | 41 | 95 | 1 | 69 | 3 | 4 | 120 | 47 |
| *Lymphatic Tissue* | 12 | 74 | 5 | 10 | 13 | 10 | 57 | 12 | 89 | 71 | 18 | 23 |
| *Pulmonary Vessel-small* | 8 | 13 | 39 | 88 | 40 | 12 | 6 | 70 | 2 | 13 | 31 | 26 |
